# Nursing intervention using healing touch in total knee replacement: A randomized controlled study protocol

**DOI:** 10.1097/MD.0000000000023735

**Published:** 2021-01-22

**Authors:** Pingfang Liu, Juan Yao, Chengfeng Qiu

**Affiliations:** a Department of nursing; b Department of nursing; c Department of Pharmacy, Huaihua First People's Hospital, Hunan, China.

**Keywords:** healing touch, total knee replacement, complication, protocol

## Abstract

**Objective:**

To assess the efficacy of healing touch (HT) for reducing pain and promoting wound healing in patients undergoing total knee replacement (TKR).

**Method:**

The experiment will be implemented from December 2020 to December 2021 and was granted through the Research Ethics Committee of Huaihua First People's Hospital (3928/823). 60 patients are included in the study. The recruitment criteria of patients includes:

1. the willingness and ability to finish the anxiety and pain questionnaires;
2. the clinical indication of TKR on the basis of medical history and physical examination; and
3. above the age of 60 years.

The exclusionary criteria includes

1. unable to cooperate with the postoperative evaluation and treatment;
2. revision of TKR;
3. diagnosis of the rheumatoid arthritis; and
4. having surgical complications.

The measurement of pain is conducted by the visual analog scale, while the levels of anxiety is measured with the State-Trait Anxiety Inventory. For all data, they are analyzed through using the software of IBM SPSS Statistics for Windows, version 20 (IBM Corp., Armonk, NY, USA).

**Results:**

Table 1 indicates the comparison of clinical outcomes between the control group and study group.

**Conclusion:**

HT appears to reduce the postoperative pain and improve patient satisfaction after TKR.

## 1 Introduction

Total knee replacement (TKR) is a kind of surgical method to treat the degenerative joint disease of knee.^[1,2]^ As the population ages, the number of TKRs increases dramatically. More than half a million TKRs were reportedly completed in the United States in 2016, which indicated the growing demand in the future.^[3]^ TKR can improve the function of knee joint and relieve pain. Nevertheless, it is related to the early postoperative pain owing to extensive soft tissue manipulation and bone resection.^[4,5]^ In addition, impaired wound healing is also a severe postoperative complications which can cause surgical site infection.^[6]^ Recently, more and more articles have concentrated on the role of nursing in TKR.^[7,8]^ Nurses are the major link in the assessment of individual impact of interventions, intervention management and pain evaluation.

Healing touch (HT) provides the nurses with a non-pharmacological technology to improve the prognosis of patients and establish contact with patients.^[9]^ The addition of biofield energy healing therapies such as HT that affect intentionally the patients energy field and environmental energy field can be incorporated into care, offering the healing atmosphere, helping to manage a variety of negative symptoms, and then generally supporting the process of healing.^[10]^ The goal of HT is to accelerate the rehabilitation process of the patient through restoring the balance of the environmental and patients energy system. It is utilized to relieve anxiety and pain, reduce depression, speed the healing of wound, promote relaxation, and increase the well-being of patients. HT is a kind of non-invasive treatment method with no known side effects. Some evidence have suggested that HT may possess some positive effects.^[11,12]^ However, few articles have introduced the HT in TKR. Thus, we conduct this randomized controlled study protocol to evaluate the influence of HT on pain relief and wound healing in patients undergoing TKR.

## 2 Method

The experiment will be implemented from December 2020 to December 2021 at Huaihua First People's Hospital. The experiment was granted through the Research Ethics Committee of Huaihua First People's Hospital (3928/823) and recorded in research registry (researchregistry6253). Before the registration, the patients who are recruited receive written informed consent. Sequence of random numbers is generated by a computer. Sequentially numbered sealed opaque envelopes are used for the concealment of random numbers. All the patients participating in this study are randomly divided into control group and study group, with 30 patients in each group.

### 2.1 Inclusion and exclusion criteria

Sixty patients are included in the study. The recruitment criteria of patients includes:

1. the willingness and ability to finish the anxiety and pain questionnaires;
2. the clinical indication of TKR on the basis of medical history and physical examination; and
3. above the age of 60 years.

The exclusionary criteria includes

1. unable to cooperate with the postoperative evaluation and treatment;
2. revision of TKR;
3. diagnosis of the rheumatoid arthritis; and
4. having surgical complications.

### 2.2 Intervention

All patients receive a fully cemented posterior stabilized TKR with patellar resurfacing. All TKRs are performed by a single orthopedic surgeon. In study group, the patients are given 4 HT sessions through 1 of 2 certified practitioners of HT. Although HT intervention aims at reducing the postoperative anxiety and pain, in HT group, the patients receive analgesic opioids in a same way as the control group patients. HT practitioner utilizes the light touch to carry out the techniques of HT. Each HT treatment lasts about 45 minutes. The first treatment is conducted immediately after the operation in postanesthesia care room. The following HT courses are performed once a day in care unit, between 2 daily physiotherapy courses, within about 2 hours after the administration of pain medication. In post-anaesthesia care room, all the patients receive the patient-controlled analgesia (PCA) at a demand morphine dose of 1 mg with an interval of 6 minutes for atresia. The follow-up adjustments of PCA setting are not limited via the research scheme. And in control group, the patients do not receive the HT courses.

### 2.3 Outcomes

The visual analogue scale is utilized for the measurement of pain and it is expressed as the 10 cm long line with “LEAST Possible Pain” on the left and “WORST Possible Pain” on the right. The levels of anxiety are measured with the State-Trait Anxiety Inventory. It is widely utilized in the clinical practice and investigation, and it is designed for the self-management. Opioid consumption is also recorded.

### 2.4 Statistical analysis

The analysis of all the data are conducted with the software of IBM SPSS Statistics for Windows, version 20 (IBM Corp., Armonk, NY, USA). Afterwards, all the data acquired are represented through the appropriate characteristics, e.g., standard deviation, and mean, median as well as percentage. And independent *t*-tests and χ^2^-tests are respectively utilized to analyze the categorical variable and continuous variable. *P* value less than .05 indicates that there is statistical significance.

## 3 Result

Table 1 indicates the comparison of clinical outcomes between the control group and study group.

**Table 1:** The comparison of clinical outcomes between the control group and study group.

Outcomes	Control group (N = 30)	Study group (N = 30)	*P* value
Pain visual analog scale at 6 hours			
Pain visual analog scale at 12 hours			
Pain visual analog scale at 24 hours			
Pain visual analog scale at 48 hours			
Opioid analgesic consumption at 6 hours			
Opioid analgesic consumption at 12 hours			
Opioid analgesic consumption at 24 hours			
Opioid analgesic consumption at 48 hours			
State-Trait Anxiety Inventory			
Length of hospitalization			
Wound complications			

## 4 Discussion

As far as we know, this is the first randomized control study aiming to assess the safety and effectiveness of HT on the pain relief and the improvement of patient satisfaction in primary TKR. With the population ages, the occurrence of knee osteoarthritis is increasing, and TKR is a successful treatment which is widely performed.^[13,14]^ The management of postoperative pain remains a fascinating topic for decades. Opioids are generally utilized as the adjunctive therapy for postoperative pain. Nevertheless, side effects associated with opioids are often occurred such as hypotension, respiratory depression, nausea and vomiting, increasing the hospital stays.^[15,16]^ Proper control of postoperative pain is beneficial to early postoperative recovery and better functional prognosis. HT is a kind of treatment initiated by nurses. It may improve the control of postoperative pain, restore the balance of the energy field of patient, and then promote the self-healing. It utilizes the non-invasive technology that uses the gentle touch and hand to clean, stimulates and balances the energy fields of human and environment, thereby affecting emotional, physical, spiritual and mental health for healing.^[17,18]^ As a complement to traditional health care, it is utilized in collaboration with other health and therapeutic approaches. This protocol may guide the subsequent randomized trial to investigate the effect of HT in patients with TKR.

## 5 Conclusion

HT appears to reduce the postoperative pain and improve patient satisfaction after TKR.

## Author contributions

Pingfang Liu plans the study design and writes the manuscript. Chengfeng Qiu reviews the protocol. Juan Yao collects data. All authors approve the submission.

**Conceptualization:** Juan Yao.

**Data curation:** Juan Yao.

**Funding acquisition:** Pingfang Liu.

**Investigation:** Chengfeng Qiu.

**Methodology:** Chengfeng Qiu.

**Writing – original draft:** Pingfang Liu.

**Writing – review & editing:** Chengfeng Qiu.
